# Hematological and hepatic effects of vascular epidermal growth factor (VEGF) used to stimulate hair growth in an animal model

**DOI:** 10.1186/1471-5945-13-15

**Published:** 2013-10-29

**Authors:** Laís Angelo Gnann, Rafael Ferreira Castro, Ligia Ajaime Azzalis, David Feder, Fabio Ferreira Perazzo, Edimar Cristiano Pereira, Paulo César Pires Rosa, Virginia Berlanga Campos Junqueira, Katya Cristina Rocha, Carlos D’ Aparecida Machado, Francisco Camargo Paschoal, Luiz Carlos de Abreu, Vitor Engrácia Valenti, Fernando Luiz Affonso Fonseca

**Affiliations:** 1 Disciplina de Farmacologia, Departamento de Morfologia, Faculdade de Medicina do ABC, Av. Príncipe de Gales, n. 821, Santo André, SP, Brazil; 2 Instituto de Ciências Ambientais, Químicas e Farmacêuticas, Departamento de Ciências Exatas e da Terra, Universidade Federal de São Paulo, Diadema, SP, Brazil; 3 Instituto de Ciências Ambientais, Químicas e Farmacêuticas, Departamento de Ciências Biológicas, Universidade Federal de São Paulo, Diadema, SP, Brazil

**Keywords:** Alopecia, Experimental model, Growth factor

## Abstract

**Background:**

Alopecia areata is the hair loss usually reversible, in sharply defined areas. The treatment of alopecia using growth factors shows interesting activity in promoting hair growth. In this concept, VEGF (vascular endothelial growth factor) is a marker of angiogenesis, stimulating hair growth by facilitating the supply of nutrients to the hair follicle, increasing follicular diameter. The aim of this study was the evaluation of a topical gel enriched with VEGF liposomes on the hair growth stimulation and its toxicological aspects.

**Methods:**

*Mesocricetus auratus* were randomly divided into three groups. Control group was treated with Aristoflex® gel, 1% group with the same gel but added 1% VEGF and 3% group with 3% VEGF. Biochemical, hematological and histological analyses were done.

**Results:**

At the end of the experiment (15th day of VEGF treatment) efficacy was determined macroscopically by hair density dermatoscopy analysis, and microscopically by hair diameter analysis. They both demonstrated that hair of the VEGF group increased faster and thicker than control. On the other hand, biochemical and hematological results had shown that VEGF was not 100% inert.

**Conclusions:**

VEGF increased hair follicle area, but more studies are necessary to confirm its toxicity.

## Background

Alopecia is a generic term to define hair loss. There are different kinds of alopecia, triggered by immunological, metabolic or unknown causes [1].

The pathologic aspect of hair loss is a social problem. Many treatments have been proposed for hair loss treatment throughout history, dating back to Egyptian papyri of 4000 a. C. Many dermatologic treatments and cosmetic products are being developed to improve hair density and to stop, or at least to decline, the loss [2].

It is very important to identify correctly which kind of alopecia will be treated to achieve a good clinical result [3]. Currently, the treatment is drawn based on clinical type of alopecia that affects the patient [4].

Two classes of active cosmetics were recently released. The first class was defined as “Growth Factors”. These proteins are produced through the process of genetic engineering, introducing the gene encoding within the DNA of *E. coli*, allowing large-scale production of these peptides. One of the great advantages of this process is that the recombinant proteins acquired in the process are 100% homologous to human, reducing the risk of allergic reactions to the product [5].

The product passes through a purification process generating the pure peptide. This peptide is then nanoencapsulated, where it is encased in liposomes, forming a protective barrier that increases the stability of the product, protecting against endogenous proteases and increasing the penetration through the skin [5].

The treatment of alopecia using growth factors shows interesting activity in promoting hair growth. In this concept, VEGF (vascular endothelial growth factor) is a marker of angiogenesis, stimulating hair growth by facilitating the supply of nutrients to the hair follicle, increasing follicular diameter [5].

It was demonstrated that the expression of VEGF in human alopecia follicles significantly decreased comparing to the normal follicles [6]. It was observed that Minoxidil, one of the pharmaceutical treatments approved for the therapy of Alopecia, could promote hair growth through upregulating the expression of VEGF in hair dermal papilla cells [7].

Considering the explanation above, the aim of this study was the quantitative evaluation of a topical gel enriched with VEGF liposomes on the hair growth stimulation and its toxicological aspects regarding changes in biochemical and hematological parameters as well as the histopathological analysis.

## Methods

### Animals

This study was approved by the Committee of Ethics and Animal Experimentation of FMABC (protocol number 001/2010). The care and handing of the animals were in accordance with the National Institute of Health guidelines.

### Treatments

18 hamsters (*Mesocricetus auratus*) were randomly divided into three groups, each one with 6 animals. Control group was treated with Aristoflex® gel, 1% group with the same gel but added 1% VEGF and 3% group with 3% VEGF. All animals had their backs shaved with a regular razor before treatment. An area of 2 cm^2^ was topically treated with 650 μL of formulation twice daily for 15 days.

On the 13th day animals were shaved once more and photos of their backs were taken with the FotoFinder dermoscope® Medicam® 500. The procedure was repeated on the 14th and 15th days. All images collected were analyzed by Dermoscope® 3.8 software.

At the end of the experiment (15^th^ day), animals were anesthetized with a mixture of xylazine (10 mg/ kg/ ip) and ketamine (100 mg/ kg/ ip). Blood was collected from the aorta artery for hematological and biochemical analyses. After animals sacrifice, the skins were collected and fixed with 10% phosphate buffered formalin.

### Hematological and biochemical analyses

Hematological analysis was conducted in the ABX Pentra 60 - Horiba® cell counter. Microscope slides were also performed for qualitative analysis of the blood cells.

Aspartate aminotransferase (AST), alanine aminotransferase (ALT), gamma glutamyl transferase (GGT) and alkaline phosphatase were assessed using Biotécnica® reagents kit.

All analytical procedures were performed following the good practice in clinical laboratory analysis.

### Histological analysis

Graded dehydration, paraffinization, and embedding were carried out on the fixed specimens. Paraffin sections (5 μm) were cut and processed for hematoxylin-eosin (HE) staining for image analysis. The images were captured with a NIKON ECLIPSE E800 microscope. Epidermal, dermal and subcutaneous thickness was measured in digital images of HE-stained sections using the Micrometrics Plus software. The thickness of hair follicles was measured in HE-stained sections at the level of the largest diameter of hair bulbs with clearly visible dermal papilla (50 hair follicles for each time point). Hair diameter was quantified by using the ImageJ® software.

### Statistical analysis

Statistical analysis was performed by using the SPSS 17.0 software. All variables were analyzed descriptively. Quantitative values were indicated by median, minimum and maximum values. For the three-group comparison, the non-parametric Kruskal-Wallis test was used, with multiple comparisons made by the Dunn’s test. A level of p < 0.05 was considered statistically significant.

## Results

### Dermoscopy images and capillary density

**Figure 1:**
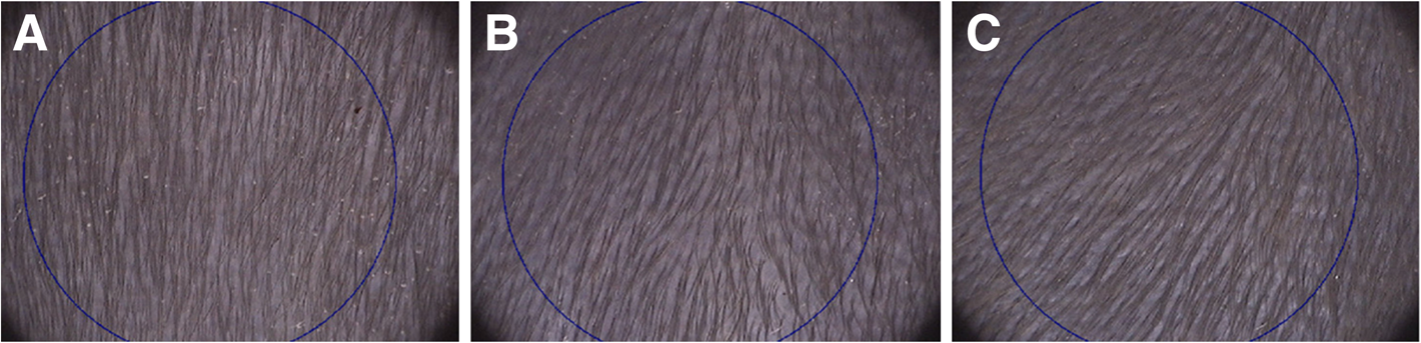
**Hair density assessed by FotoFinder dermoscope® Medicam® 500. (A)** Control group. **(B)** 1% VEGF. **(C)** 3% VEGF.

FotoFinder dermoscope® Medicam® 500 and Dermoscope® 3.8 software were used to analyze the hair density of the three groups: control, 1% VEGF and 3% of VEGF after 15 days of treatment. The Figure 1 shows hair density assessed by FotoFinder dermoscope® Medicam® 500. The Table 1 shows analyzed area: 0.65 cm2; density unit: 1/cm2.

**Table 1:** **Hair density obtained by dermatoscopy analysis (analyzed area: 0.65 cm**^**2**^**; density unit: 1/cm**^**2**^**)**

***Group***	***Average density (1/ cm***^***2***^ ***)***	***s.d.***	***Min.***	***Max.***	p*
Control	387.93	59.66	312.6	493.1	0.999
1% VEGF	388.57	64.45	305.7	506.9	0.9525
3% VEGF	379.28	49.6	207.4	445.5	0.002

*p<0.05, N = 6. Non-parametric Kruskal-Wallis test, with multiple comparisons made by the Dunn’s test for the three-groups comparison (control x 1% x 3%).

### Histological analysis

**Figure 2:**
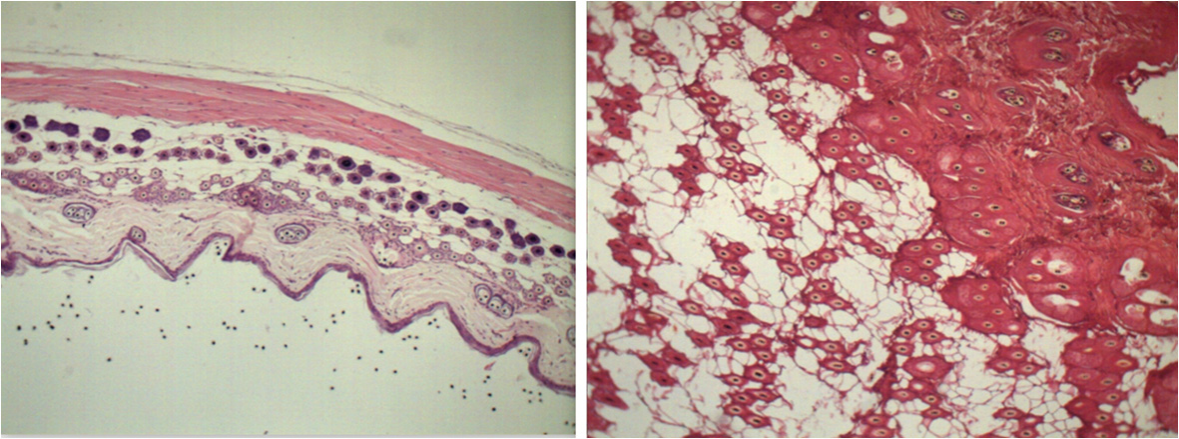
Cross and longitudinal sections of animal treated with 1% of VEGF.

**Figure 3:**
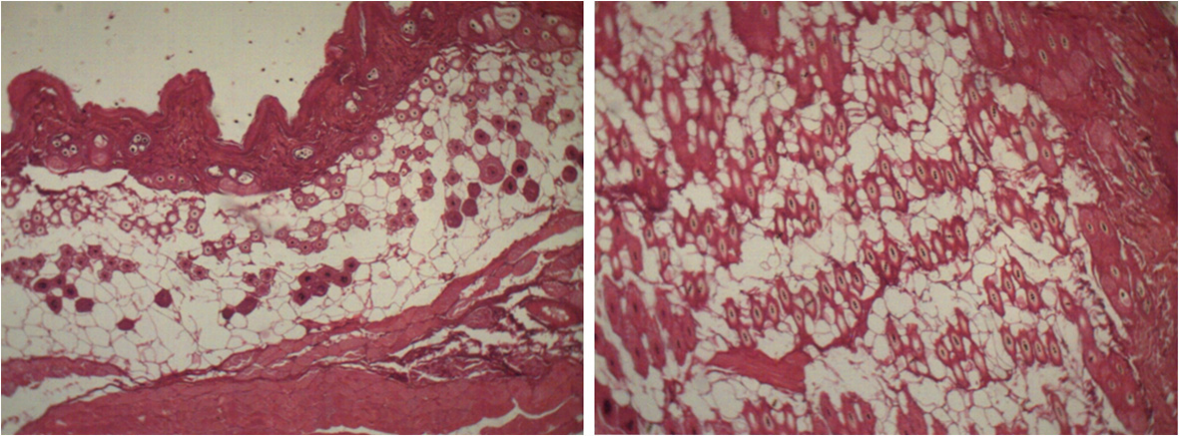
Cross and longitudinal sections of animal treated with 3% of VEGF.

**Figure 4:**
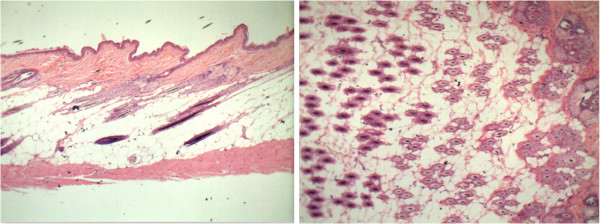
Cross and longitudinal sections of control group.

Photos were taken under a digital microscope and analyzed by ImageJ®, measuring the area of each hair follicle in order to compare the area of the hair follicle of the control group, 1% VEGF and 3% VEGF (Table 2, Figures 2, 3, and 4).

**Table 2:** **Hair density obtained by ImageJ® software analysis (analyzed area: 0.65 cm**^**2**^**; density unit: 1/cm**^**2**^**)**

***Color***	***Group***	***Average Area***	***s.d.***	***Min.***	***Max.***	p**
	Control	559.21	543.36	54	6980	0.996
HE	1% VEGF	613.8	686.86	52	17253	0.001
	3% VEGF	707.77	914.52	48	22116	0.001

**p<0.05, N = 6. Non-parametric Kruskal-Wallis test, with multiple comparisons made by the Dunn’s test for the three-groups comparison (control x 1% x 3%).

Biochemical and hematological results (Tables 3 and 4).

**Table 3:** Biochemical analysis

***Group***	***Biochemical parameter***	***Average (U/L)***	***s.d.***	***p***
Control	GGT	17.6	10.64	0.2453
1% VEGF	GGT	3	0	
3% VEGF	GGT	6	7.07	
Control	AST	91.5	18.59	0.2834
1% VEGF	AST	130.83	160.06	
3% VEGF	AST	70.7	30.22	
Control	ALT	194.7	84.57	0.5195
1% VEGF	ALT	270	342.69	
3% VEGF	ALT	139.2	38.45	
Control	Alkaline phosphatase	51	16.87	0.9439
1% VEGF	Alkaline phosphatase	77.5	86.07	
3% VEGF	Alkaline phosphatase	96.3	84.32	

GGT, gamma-glutamyl transferase; AST, aspartate amino transferase; ALT, alanine amino transferase. Non-parametric Kruskal-Wallis test, with multiple comparisons made by the Dunn’s test for the three-groups comparison (control x 1% x 3%). ^*^A level of p < 0.05 was considered statistically significant.

**Table 4:** Hematological analysis

***Variable***	***Group***	***n***	***Average***	***s.d.***	***Min.***	***Max.***	p*
WBC (10^3^/mm^3^)	Control	4	4.2	1.13	3.1	5.7	0.3834
	1%	6	3.2	0.93	2.1	4.9	
	3%	6	3.4	0.86	2	4.5	
RBC (10^6^/mm^3^)	Control	4	8.94	0.36	8.63	9.4	0.0217
	1%	6	8.12	0.72	6.69	8.59	
	3%	6	8.57	0.32	8.23	9.14	
Hb (g/dL)	Control	4	16	0.88	15.4	17.3	0.1649
	1%	6	15.3	0.66	14	15.8	
	3%	6	15.4	0.6	14.8	16.5	
Ht (%)	Control	4	47.6	2.01	45.2	49.7	0.1253
	1%	6	43.4	3.94	35.5	45.6	
	3%	6	45.8	2.1	44	49.5	
MCV (L/μm^3^)	Control	4	53	0.96	52	54	0.9311
	1%	6	53.5	0.55	53	54	
	3%	6	53.3	0.82	52	54	
MCH (L/pg)	Control	4	17.7	0.69	16.8	18	0.0201
	1%	6	18.9	1.03	18	20.9	
	3%	6	17.9	0.18	17.7	18.2	
Plt (10^3^/mm^3^)	Control	4	527	150	353	712	0.5584
	1%	6	451	114.4	321	603	
	3%	6	482	67.6	402	536	
Lin (%)	Control	4	70.5	4.85	63.4	74.3	0.0615
	1%	6	77.4	3.12	72.8	81.7	
	3%	6	70.3	9.33	62.4	81.5	
Mon (%)	Control	4	0.2	0.05	0.1	0.2	0.5601
	1%	6	0.2	0.1	0	0.2	
	3%	6	1.05	0.6	0	1.5	
Eos (%)	Control	4	1.2	0.5	0.5	1.6	0.018
	1%	6	0.3	0.4	0	1.1	
	3%	6	1.18	1.04	0.4	3.2	
Bas (%)	Control	4	4.5	1.9	2.1	7	0.6479
	1%	6	5.6	1.8	3.1	7.6	
	3%	6	4.03	2.39	1.3	8	

WBC, Leucocytes; RBC, Erythrocytes; Hb, Haemoglobin; Ht, Haematocrit; MCV, Mean corpuscular volume; MCH, Mean corpuscular haemoglobin; Plt, Platelets; Lin, Lymphocytes; Mon, Monocytes; Eos, Eosinophils; Bas, Basophils. Non-parametric Kruskal-Wallis test, with multiple comparisons made by the Dunn’s test. *A level of p < 0.05 was considered statistically significant.

## Discussion

Our previous results have shown that a liposomal liquid gel formulation containing insulin-like growth factor-1 (IGF-1) was efficacious in promoting hair growth and density, without evidence of adverse effects such as hepatoxicity [8]. This present study aimed to verify the safety and efficacy of another growth factor – VEGF. *Mesocricetus auratus* was selected as experimental animal in both studies because growth factors would be readily absorbed reaching systemic circulation after its topical application. Thus, this could be a suitable animal model to study efficacy and safety of IGF-1 and VEGF [9].

The use of animal models has many advantages since the follicle are in their natural physiological environment and undergo normal cyclic activity. Another popular model is the black mouse C57BL/6. Unfortunately, mice have the significant drawback of patchy growth once the second wave of hair growth has been completed [10]. Furthermore, FotoFinder dermoscope Medicam® 500 needs a larger area to analyze the hair density.

Our results show that as higher the concentration of VEGF present in the gel sample, bigger is the hair follicle area. Yano et al. [11] identified VEGF as a major mediator of hair follicle growth and cycling and provided the first direct evidence that improving follicle vascularization promoted hair growth and increased hair follicle and hair size in mice. It is known that VEGF is a growth factor that stimulates vasculogenesis and angiogenesis, stimulating hair growth by facilitating the supply of nutrients to the hair follicle, providing even an increase in the base of the follicle diameter [5,12,13].

Biochemical and hematological results have shown that VEGF is not 100% inert. Data obtained and represented in the Table 3 have indicated that the use of VEGF enhanced AST and ALT levels in the 1% VEGF group. Alkaline phosphatase values were high in both 1% and 3% VEGF groups. Despite all results were not significant, they should be continuously analyzed.

VEGF-treated animals showed lower red blood cells and mean corpuscular hemoglobin values than control animals (Table 4). No significant differences were observed when other blood parameters were analyzed, except to eosinophils. VEGF treatment diminished the number of eosinophils.

The treatment of alopecia using growth factors shows interesting activity in promoting hair growth. On the other hand, more toxicological studies are necessary to confirm their safety.

## Conclusion

VEGF increased hair follicle area, but more studies are necessary to confirm its toxicity. As far as we know, this is the first reference in literature that associates hematological and hepatic effects with VEGF used to promote hair growth.

## Abbreviations

VEGF: vascular epidermal growth factor.

## Competing interests

Authors declare there are no competing interests.

## Authors’ contribution

LAG and RFC carried out the hematological and the histological studies. LAA drafted the manuscript. DF performed the statistical analysis. FFP, ECP, PCPR contributed to the acquisition of data. VBCJ, KCR, CDAM and FCP contributed to analysis and interpretation of data. LCA and VEV revised the manuscript critically. FLAF conceived of the study, and participated in its design and coordination and helped to draft the manuscript. All authors read and approved the final manuscript.

## Pre-publication history

The pre-publication history for this paper can be accessed here:

http://www.biomedcentral.com/1471-5945/13/15/prepub

## References

[B1] Tosti A Piraccini BM van Neste DJ Telogen effluvium after allergic contact dermatitis of the scalp Arch Dermatol 2001 137 187 190 11176691

[B2] Shapiro J Price V Hair regrowth: therapeutic agents Dermatol Clin 1998 16 341 356 10.1016/S0733-8635(05)70017-6 9589208

[B3] Rivitti EA Alopecia areata: revisão e atualização An Bras Dermatol 2005 80 57 58

[B4] Maia CP Fernandes NC Tratamento da alopecia areata com corticóide tópico: Estudo prospectivo randomizado duplo cego em crianças An Bras Dermatol 2003 78 63 71

[B5] Ozeki M Tabata Y In vivo promoted growth of mice hair follicles by the controlled release of growth factors Biomaterials 2003 24 2387 94 10.1016/S0142-9612(03)00045-0 12699676

[B6] Goldman CK Tsai JC Soroceanu L Gillespie GY Loss of vascular endothelial growth factor in human alopecia hair follicles J Invest Dermatol 1995 104 5 18S 20S 10.1038/jid.1995.40 7738377

[B7] Messenger AG Rundegren J Minoxidil: mechanisms of action on hair growth Br J Dermatol 2004 150 186 94 10.1111/j.1365-2133.2004.05785.x 14996087

[B8] Castro RF Azzalis LA Feder D Safety and efficacy analysis of liposomal insulin-like growth factor-1 in a fluid gel formulation for hair-loss treatment in a hamster model Clin Exp Dermatol 2012 37 909 912 10.1111/j.1365-2230.2012.04441.x 22924775

[B9] Mitchell LH Johnson TR Lu GW Rational design of a topical androgen receptor antagonist for the suppression of sebum production with properties suitable for follicular delivery J Med Chem 2010 53 4422 4427 10.1021/jm9018004 20462217

[B10] Randall VA Sundberg JP Philpott MP Animal and in vitro models for the study of hair follicles JID Symposium Proceedings 2003 8 39 45 10.1046/j.1523-1747.2003.12170.x 12894993

[B11] Yano K Brown LF Detmar M Control of hair growth and follicle size by VEGF-mediated angiogenesis J Clin Invest 2001 107 409 417 10.1172/JCI11317 11181640 PMC199257

[B12] Bartels NG Jahnke I Patzelt A Hair shaft abnormalities in alopecia areata evaluated by optical coherence tomography Skin Res Technol 2011 17 201 205 10.1111/j.1600-0846.2010.00484.x 21241369

[B13] Kim MJ Lim C Lee JY Visible-to-near IR quantum dot-based hypermulticolor high-content screening of herbal medicines for the efficacy monitoring of hair growth promoting and hair loss inhibition J Biomol Screen 2013 18 462 73 10.1177/1087057112464574 23190736

